# Hemorrhage Risk Among Patients With Breast Cancer Receiving Concurrent Direct Oral Anticoagulants With Tamoxifen vs Aromatase Inhibitors

**DOI:** 10.1001/jamanetworkopen.2022.19128

**Published:** 2022-06-28

**Authors:** Tzu-Fei Wang, Anna E. Clarke, Arif A. Awan, Peter Tanuseputro, Marc Carrier, Manish M. Sood

**Affiliations:** 1 Department of Medicine, University of Ottawa at the Ottawa Hospital, Ottawa, Ontario, Canada; 2 Clinical Epidemiology Program, Ottawa Hospital Research Institute, Ottawa, Ontario, Canada; 3 ICES uOttawa, Toronto, Ontario, Canada; 4 Bruyère Research Institute, Ottawa, Ontario, Canada

## Abstract

**Importance:**

Tamoxifen is commonly used as adjuvant therapy in breast cancer and is proposed to interfere with cytochrome P450 enzyme and P-glycoprotein pathways. Concurrent use with direct oral anticoagulants (DOACs) poses the threat of a potentially dangerous drug-drug interaction by leading to an increase in hemorrhage risk.

**Objective:**

To assess the risk of hemorrhage in patients with breast cancer coprescribed a DOAC and tamoxifen compared with a DOAC and an aromatase inhibitor (AI).

**Design, Setting, and Participants:**

This population-based, retrospective cohort study was conducted among adults aged 66 years or older who were prescribed tamoxifen (compared with an AI) concurrently with a DOAC in Ontario, Canada, between June 23, 2009, and November 30, 2020, and followed up until December 31, 2020.

**Interventions:**

Concurrent prescription of a DOAC and tamoxifen compared with a DOAC and an AI.

**Main Outcomes and Measures:**

The primary outcome was major hemorrhage requiring an emergency department visit or hospitalization after prescription. Overlap weighted Cox proportional hazards models, accounting for multiple covariates, were used to assess the association between hemorrhage and tamoxifen or AI use with a DOAC.

**Results:**

Among a total of 4753 patients (4679 [98.4%] women; mean [SD] age, 77.4 [7.4] years), 1179 (24.8%) were prescribed tamoxifen, and 3574 (75.2%) were prescribed an AI. Rivaroxaban (2530 [53.2%]) and apixaban (1665 [35.0%]) were the most frequently used DOACs. Patients taking AIs were younger than patients taking tamoxifen (mean [SD] age, 77.1 [7.3] vs 78.3 [7.6] years), with higher Charlson Comorbidity Index (mean [SD], 1.8 [2.4] vs 1.5 [2.2]) and more advanced cancer stage (stages III and IV, 569 [15.9%] vs 127 [10.8%]). During a median follow-up of 166 days (IQR, 111-527 days), tamoxifen was not associated with a higher risk of major hemorrhage (29 of 1179 [2.5%]) compared with an AI (119 of 3574 [3.3%]) when combined with a DOAC (absolute risk difference, −0.8%; weighted hazard ratio, 0.68 [95% CI, 0.44-1.06]). These results were similar in additional analyses using a more liberal definition of hemorrhage, accounting for kidney function, limiting follow-up to 90 days, stratifying by incident and prevalent DOAC users, and accounting for cancer duration and the competing risk of death.

**Conclusions and Relevance:**

In this cohort study, findings suggest that among DOAC users, the concurrent use of tamoxifen was not associated with a higher risk of hemorrhage compared with the concurrent use of an AI. These findings should directly inform prescribers regarding the apparent safety of concurrent DOAC and tamoxifen use.

## Key Points

**Question:**

Is the concurrent use of tamoxifen and a direct oral anticoagulant (DOAC) associated with important drug-drug interactions and an increased risk of hemorrhage?

**Findings:**

In this large population-based cohort study including 4753 adult patients with breast cancer, tamoxifen combined with a DOAC was not associated with a higher risk of major hemorrhage requiring an emergency department visit or hospitalization compared with the combination of an aromatase inhibitor and a DOAC.

**Meaning:**

These findings suggest that among DOAC users, the concurrent use of tamoxifen is not associated with a higher risk of hemorrhage compared with concurrent use of aromatase inhibitors.

## Teaser

This cohort study evaluates the risk of hemorrhage requiring hospitalization or emergency department visits in patients with breast cancer taking direct oral anticoagulants (DOACs) concurrently with tamoxifen compared with an aromatase inhibitor.

## Introduction

Venous thromboembolism (VTE) is a known complication associated with cancer. Patients with cancer are at a 12-fold higher risk of VTE compared with those without cancer, and this risk increases to 23-fold with cancer therapy.^1^ In addition, patients with cancer are at a 2-fold elevated risk of atrial fibrillation (AF) and arterial thromboses compared with the general population.^2,3^ Thus, anticoagulation is frequently required despite the 2-fold elevated risk of hemorrhage in patients with cancer compared with those without cancer.^4^ Hemorrhagic events are significant and can lead to invasive examinations, hospitalizations, and health care costs.^5,6^ Therefore, safe and effective anticoagulant prescribing practices for patients with cancer are critically important.

Direct oral anticoagulants (DOACs) are becoming the preferred anticoagulant for patients with cancer for the prevention and treatment of thromboembolism.^7,8,9^ Direct oral anticoagulants are substrates of 2 main metabolic pathways: P-glycoprotein (P-gp) cell transporters and cytochrome P450 enzyme (CYP3A4) in the liver.^10^ Edoxaban and dabigatran are involved in the P-gp pathway, whereas apixaban and rivaroxaban are involved in both. Concomitant medications that inhibit P-gp and/or CYP3A4 pathways can lead to higher DOAC concentrations and a heightened risk of hemorrhage, largely based on in vitro pharmacokinetic data.^11,12^ Major international guidelines, product monographs, and consensus statements suggest caution when using DOACs with potentially strong interfering drugs in patients with cancer.^7,8^ However, whether this results in clinically relevant bleeding events remains largely unknown.

Breast cancer is the most common nonskin cancer in women, accounting for 25% of new female cancer cases in 2020.^13^ It is associated with a heightened risk of thromboembolic events (annual VTE risk, 2%-6%)^14^ and a 2-fold risk of AF; thus, patients with breast cancer commonly use anticoagulation therapy.^15,16^ Tamoxifen, used for hormone receptor–positive breast cancer, is reported as an inhibitor in the CYP3A4 and P-gp pathways,^17,18,19^ posing a possible drug-drug interaction (DDI) with DOACs and heightened bleeding risk.^20^ Because clinical evidence of this proposed drug interaction is limited, we evaluated the risk of hemorrhage requiring hospitalization or emergency department visits in patients with breast cancer taking DOACs with tamoxifen compared with the concurrent use of DOACs and an aromatase inhibitor (AI), an active comparator that is also used to treat hormone receptor–positive breast cancer but is not involved in CYP3A4 or P-gp pathways.

## Methods

### Data Sources

In this population-based retrospective cohort study, we used deidentified linked electronic health care databases held in ICES, an independent, nonprofit research organization that houses multiple linked databases with population-based health and social data in Ontario (eTable 1 in the Supplement). Ontario is the largest province in Canada, with a population of 14.5 million, with medical care reimbursed through a single government-funded health insurance system. The Ontario Drug Benefit (ODB) Database contains comprehensive oral prescriptions for individuals 65 years or older. Medication claims include information on the drug identification number, trade name, therapeutic class, pill strength, quantity dispensed, and number of days supplied with an error rate of 0.7%.^21^ Records were linked to other health administrative databases that were used to capture the occurrence of major bleeding or thrombotic events. Hospitalization data were obtained from the Discharge Abstract Database, which contains clinical and demographic data. Emergency department visits were identified through the National Ambulatory Care Reporting System. The Ontario Cancer Registry, which captures more than 95% of Ontario pathology reports, was used to identify individuals who received a diagnosis of breast cancer.^22^ These data sets were linked using unique encoded identifiers and analyzed at ICES. The use of ICES data was authorized under section 45 of Ontario’s Personal Health Information Protection Act,^23^ and additional review by the ethics board was not required. All data used were deidentified, and patient informed consent was waived as per the Ontario Ministry of Health. We report our findings according to the recommended Reporting of Studies Conducted Using Observational Routinely Collected Health Data (RECORD) statement and the Strengthening the Reporting of Observational Studies in Epidemiology (STROBE) reporting guideline.

### Study Cohort

The study population included older adults (≥66 years) who had filled an outpatient prescription for a DOAC (rivaroxaban, apixaban, dabigatran, or edoxaban) and a cancer drug of interest (tamoxifen, anastrozole, letrozole, or exemestane) (eTable 2 in the Supplement) between June 23, 2009 (when DOACs became available in Ontario), and November 30, 2020, in Ontario, Canada, and had a breast cancer diagnosis in the Ontario Cancer Registry during or within 5 years before concurrent use. The index date to enter the cohort was the date of concurrent use of a DOAC and tamoxifen or an AI. In Ontario, the filling of prescription drugs is captured for individuals 65 years or older under the universal Ontario Drug Benefit for seniors. As such, an age cutoff of 66 years was chosen to allow a minimum 1-year look-back period for medication prescribing. Patients were excluded if they (1) had invalid data or data that could not be linked; (2) were non-Ontario residents; (3) were younger than 66 years at the index date; (4) had a concurrent prescription of an anticoagulant other than a DOAC; (5) had at least 1 prescription of any strong CYP3A4 or P-gp inhibitor within 90 days before the index date (eTable 3 in the Supplement); (6) had a concurrent prescription of a CDK4/6 inhibitor (palbociclib, ribociclib, or abemaciclib) with tamoxifen or an AI, given that these medications could result in additional DDI^24,25^; (7) received chronic dialysis or had a kidney transplant before the index date; (8) had an index date and stop date on the same day; or (9) switched tamoxifen or AIs within 1 year before the index date (cohort flow diagram is found in eFigure 1 in the Supplement).

### Study Design

We conducted a retrospective cohort study to compare the association of clinically relevant hemorrhage (leading to hospitalization and/or emergency department visit) in patients receiving a concurrent DOAC and tamoxifen compared with a concurrent DOAC and an AI. Confounders considered included age; sex; index year; income; residence in a long-term care facility; rural residence; previous hemorrhage; comorbidities, such as history of hypertension, diabetes, stroke or transient ischemic attacks, AF, myocardial infarction, heart failure, coronary artery disease, angina, coronary artery bypass graft surgery, percutaneous coronary intervention, acute coronary syndrome, peripheral vascular disease, VTE, liver disease, or chronic kidney disease; Charlson Comorbidity Index^26^; stage of breast cancer at index date; and other concurrent medications, including angiotensin-converting enzyme inhibitors or angiotensin II receptor blockers, calcium channel blockers, β-blockers, agents to lower lipid levels, nonsteroidal anti-inflammatory drugs, proton pump inhibitors, antiplatelet agents, and selective serotonin reuptake inhibitors. We considered different types, doses, and duration of DOAC and cancer drug therapies in the analyses.

### Outcomes

The primary outcome was a major hemorrhage requiring an emergency department visit or hospitalization, including an upper or lower gastrointestinal tract hemorrhage or an intracerebral, subarachnoid, or other nontraumatic intracranial hemorrhage. Our validated definition of hemorrhage used *International Statistical Classification of Diseases and Related Health Problems, Tenth Revision* (*ICD-10*), codes in the Canadian Institute for Health Information Discharge Abstract Database and National Ambulatory Care Reporting System (eTable 4 in the Supplement). Prior similar studies have shown that these codes had a positive predictive value of 87% and a negative predictive value of 92%.^27^ We further examined a more liberal definition of hemorrhage that included any bleeding event or receipt of a blood transfusion associated with an emergency department visit or hospitalization.

Outcomes were analyzed as treated, for which patients were censored when they stopped or switched to another hormonal or anticoagulant therapy. Individuals were followed up until the primary study outcome, death, loss of Ontario health insurance eligibility, or the end of the follow-up period on December 31, 2020.

### Statistical Analysis

Descriptive statistics were used to compare the distribution of factors and outcomes. Mean (SD) values were calculated for variables with continuous distributions (or median values [IQRs], if skewed), and frequencies and proportions for categorical variables. Standardized differences were used to compare characteristics between groups, by describing differences between group means relative to the pooled SD, and a significant difference was considered to be greater than 0.100.^28^ We used overlap weighting based on the propensity score to adjust for differences in all baseline characteristics listed in Table 1. A propensity score for exposure (tamoxifen) was calculated and applied in a weighted analysis (overlap weights method), with each patient’s weight assigned proportionally to the probability of that patient being allocated to the alternative group (AI group).^29,30^ The overlap weighting method assigns less weight to observations with extreme propensity scores and accounts for all variables without excluding study participants. Missing data were accounted for by multiple imputations. Specifically, rurality (10 [0.2%] missing), neighborhood-level income quintile (16 [0.3%] missing), and cancer stage (381 [8.0%] missing) were imputed 5 times and incorporated in the modeling. After assessing the stability of the results across imputations, the first imputation was used for graphing (eFigure 2 in the Supplement). Weighted Cox proportional hazards models were used to assess the association of hemorrhage and tamoxifen or AI use with a DOAC. We included type of DOAC as an interaction term to evaluate the association of different DOACs with hemorrhage. All analyses were conducted using SAS Enterprise Guide, version 7.1 (SAS Institute, Inc); 95% CIs that did not overlap with 1 were treated as statistically significant. We calculated *P* values using *t* test statistics for the analysis of interaction by DOAC type and log-rank tests to compare Kaplan-Meier curves (eFigures 3 and 4 in the Supplement); 2-sided *P* < .05 was considered statistically significant.

**Table 1.** Baseline Characteristics of Patients With Breast Cancer Receiving DOACs and Tamoxifen Compared With Aromatase Inhibitors

Characteristic	Patient treatment group, No. (%)^a^		Standardized difference^b^	
	Tamoxifen (n = 1179)	Aromatase inhibitors (n = 3574)	Before weighting	After weighting
Age, mean (SD), y	78.3 (7.6)	77.1 (7.3)	0.159	0
Sex				
Female	1111 (94.2)	3568 (99.8)	0.335	0
Male	68 (5.8)	6 (0.2)	0.335	0
Index year				
2009-2010	9 (0.8)	33 (0.9)	0.017	0
2011	12 (1.0)	47 (1.3)	0.028	0
2012	37 (3.1)	174 (4.9)	0.088	0
2013	72 (6.1)	199 (5.6)	0.023	0
2014	104 (8.8)	291 (8.1)	0.024	0
2015	116 (9.8)	337 (9.4)	0.014	0
2016	160 (13.6)	404 (11.3)	0.069	0
2017	176 (14.9)	417 (11.7)	0.096	0
2018	180 (15.3)	558 (15.6)	0.010	0
2019	181 (15.3)	600 (16.8)	0.039	0
2020	132 (11.2)	514 (14.4)	0.095	0
Nearest census-based neighborhood income quintile				
1 (Low)	233 (19.8)	731 (20.5)	0.017	0
2	225 (19.1)	790 (22.2)	0.075	0
3	207 (17.6)	687 (19.3)	0.043	0
4	245 (20.9)	666 (18.7)	0.054	0
5 (High)	265 (22.5)	688 (19.3)	0.079	0
Rural residence	163 (13.8)	442 (12.4)	0.044	0
LTC residence	36 (3.1)	109 (3.0)	0	0
Comorbidities				
Major hemorrhage within 1 y prior	22 (1.9)	78 (2.2)	0.022	0
Any hemorrhage within 1 y prior	39 (3.3)	130 (3.6)	0.018	0
Hypertension	921 (78.1)	2825 (79.0)	0.023	0
Diabetes	351 (29.8)	1198 (33.5)	0.081	0
Stroke or TIA	81 (6.9)	325 (9.1)	0.082	0
Atrial fibrillation	387 (32.8)	1257 (35.2)	0.050	0
MI	46 (3.9)	120 (3.3)	0.029	0
Heart failure	253 (21.5)	777 (21.7)	0.007	0
CAD	327 (27.7)	898 (25.1)	0.059	0
Angina	28 (2.4)	103 (2.9)	0.032	0
CABG	10 (0.8)	13 (0.4)	0.062	0
PCI	28 (2.4)	72 (2.0)	0.025	0
Acute coronary syndrome	334 (28.3)	932 (26.1)	0.051	0
Peripheral vascular disease	15 (1.3)	44 (1.2)	0.004	0
VTE	274 (23.2)	738 (20.6)	0.063	0
Liver disease	54 (4.6)	159 (4.4)	0.006	0
CKD	309 (26.2)	946 (26.5)	0.006	0
Charlson Comorbidity Index, mean (SD)	1.5 (2.2)	1.8 (2.4)	0.127	0
Breast cancer stage				
I	549 (46.6)	1491 (41.7)	0.098	0
II	409 (34.7)	1227 (34.3)	0.008	0
III	106 (9.0)	424 (11.9)	0.094	0
IV	21 (1.8)	145 (4.1)	0.136	0
Missing	94 (8.0)	287 (8.0)	0.002	0
Medications				
ACE or ARB	617 (52.3)	1947 (54.5)	0.043	0
Calcium channel blockers	394 (33.4)	1364 (38.2)	0.099	0
Beta-blocker	514 (43.6)	1668 (46.7)	0.062	0
Lipid-lowering agents	576 (48.9)	1916 (53.6)	0.095	0
NSAIDs	299 (25.4)	888 (24.8)	0.012	0
Proton pump inhibitors	518 (43.9)	1587 (44.4)	0.009	0
Antiplatelet agents	64 (5.4)	210 (5.9)	0.019	0
SSRIs	205 (17.4)	650 (18.2)	0.021	0
DOAC type				
Dabigatran	127 (10.8)	375 (10.5)	0.009	0
Rivaroxaban	625 (53.0)	1905 (53.3)	0.006	0
Apixaban	421 (35.7)	1244 (34.8)	0.019	0
Edoxaban	6 (0.5)	50 (1.4)	0.092	0
DOAC daily dose, mean (SD), mg				
Dabigatran	251.3 (42.3)	257.5 (84.4)	0.092	0
Rivaroxaban	16.5 (7.4)	16.7 (14.2)	0.015	0
Apixaban	8.6 (4.0)	8.7 (3.4)	0.007	0
Edoxaban	50.0 (15.5)	54.4 (15.8)	0.281	0

Abbreviations: ACE, angiotensin-converting enzyme inhibitor; ARB, angiotensin II receptor blocker; CABG, coronary artery bypass graft surgery; CAD, coronary artery disease; CKD, chronic kidney disease; DOAC, direct oral anticoagulant; LTC, long-term care; MI, myocardial infarction; NSAIDs, nonsteroidal anti-inflammatory drugs; PCI, percutaneous coronary intervention; SSRI, selective serotonin reuptake inhibitor; TIA, transient ischemic attack; VTE, venous thromboembolism.

^a^ Unless otherwise indicated, data are expressed as number (%) of patients. Percentages have been rounded and may not total 100. Owing to missing data, numbers may not total numbers in column headings.

^b^ Standardized difference of greater than 0.100 indicates significant differences between the 2 groups.

We conducted several additional analyses to evaluate for the consistency of our findings. First, we restricted analyses to those with an estimated glomerular filtration rate (eGFR) measure and further added eGFR in our models. Second, we limited our outcome ascertainment window to the first 90 days after concurrent prescription. Third, we stratified by new DOAC users (individuals who initiated DOAC therapy while receiving tamoxifen or AIs) vs prevalent DOAC users (individuals who initiated tamoxifen or AI therapy while receiving a DOAC) because the risk of hemorrhage may differ. Fourth, we adjusted our models for duration from cancer diagnosis. Fifth, we examined death as a competing risk. Last, we assessed for residual confounding by using a composite of cholecystitis, diverticulitis, and appendicitis as negative dummy outcomes, for which we expected no association with drug use (eTable 4 in the Supplement).

## Results

During the study period (June 23, 2009, to November 30, 2020), among a total of 539 366 patients prescribed DOACs, 4753 who were 66 years or older had a diagnosis of breast cancer and received tamoxifen or an AI. Of these, 4679 patients (98.4%) were women, and 74 patients (1.6%) were men; the mean (SD) age was 77.4 (7.4) years. Race and ethnicity data were not available in the database so could not be included in the analysis. In this cohort, 1179 patients received tamoxifen, and 3574 received an AI (1865 [52.2%] letrozole, 1581 [44.2%] anastrozole, and 128 [3.6%] exemestane). Prescriptions for DOACs included rivaroxaban for 2530 patients (53.2%), apixaban for 1665 patients (35.0%), dabigatran for 502 patients (10.6%), and edoxaban for 56 patients (1.2%) (see eFigure 1 in the Supplement for cohort flow diagram). The use of concurrent DOAC and hormone therapy increased over time and similarly for both tamoxifen and AIs. The most common comorbid conditions included hypertension (3746 [78.8%]), AF (1644 [34.6%]), and diabetes (1549 [32.6%]), whereas 1012 patients (21.3%) had a history of VTE. Most patients had stage I or II disease (3676 [77.3%]), whereas 166 patients (3.5%) had metastatic disease. The most common other concurrent medications included angiotensin-converting enzyme inhibitors and/or angiotensin II receptor blockers (2564 [53.9%]) and agents to lower lipid levels (2492 [52.4%]). One-quarter of patients (1187 [25.0%]) were taking nonsteroidal anti-inflammatory drugs, whereas another 274 (5.8%) took antiplatelet agents concomitantly. Table 1 lists the baseline characteristics according to hormonal agents before and after overlap weighting. Patients taking AIs vs tamoxifen included more women (3568 [99.8%] vs 1111 [94.2%]), were younger (mean [SD] age, 77.1 [7.3] vs 78.3 [7.6] years), had a higher Charlson Comorbidity Index (mean [SD], 1.8 [2.4] vs 1.5 [2.2]), and had more advanced stage cancer (stages III and IV, 569 [15.9%] vs 127 [10.8%]). After overlap weighting, the standard differences all reached 0 (Table 1 and eFigure 2 in the Supplement), indicating successful adjustment for all listed baseline characteristics.

During a median follow-up of 166 days (IQR, 111-527 days), a total of 148 patients (3.1%) had major hemorrhage events, corresponding to 29.2 (95% CI, 24.9-34.3) per 1000 person-years: 131 of these (88.5%) had GI tract bleeding and 17 (11.5%) had brain bleeding. The risk of major hemorrhage while receiving a DOAC was not higher with concurrent tamoxifen (29 of 1179 patients [2.5%]; 23.4 [95% CI, 16.3-33.7] per 1000 person-years) compared with a concurrent AI (119 of 3574 patients [3.3%]; 31.1 [95% CI, 26.0-37.2] per 1000 person-years). This was consistent in weighted models (absolute risk difference, −0.8%; hazard ratio [HR], 0.68 [95% CI, 0.44-1.06]) (Table 2 and eFigure 3 in the Supplement). When we used a more liberal definition of hemorrhage, including any bleeding events or receipt of a blood transfusion, 223 patients (4.7%) had a bleeding event, including 58 of 1179 (4.9%) receiving tamoxifen (47.7 [95% CI, 36.9-61.8] per 1000 person-years) and 165 of 3574 (4.6%) receiving an AI (43.7 [95% CI, 37.5-50.9] per 1000 person-years; weighted HR, 1.04 [95% CI, 0.75-1.43]) (Table 2 and eFigure 4 in the Supplement). These events included GI tract bleeding in 127 patients, brain bleeding in 17, and bleeding events in the genitourinary, gynecological, joint, eye, respiratory tract, or nonspecific sites in 81 (some had bleeding in ≥1 site). No difference in major or all hemorrhage was found by DOAC type (*P* = .29 and *P* = .90, respectively, for interaction).

**Table 2.** Rates of Hemorrhage Comparing Use of Tamoxifen and Aromatase Inhibitors in Patients Receiving Concurrent Direct Oral Anticoagulants

Characteristic	No./total No.	Cumulative incidence, %	Rate per 1000 person-years (95% CI)	Weighted HR (95% CI)
Major hemorrhage				
Tamoxifen	29/1179	2.5	23.4 (16.3-33.7)	0.68 (0.44-1.06)
Aromatase inhibitors	119/3574	3.3	31.1 (26.0-37.2)	
Any hemorrhage				
Tamoxifen	58/1179	4.9	47.7 (36.9-61.8)	1.04 (0.75-1.43)
Aromatase inhibitors	165/3574	4.6	43.7 (37.5-50.9)	

Abbreviation: HR, hazard ratio.

Tamoxifen was not associated with a higher hemorrhage risk in several additional analyses, including accounting for eGFR, limiting the follow-up window to 90 days, accounting for incident and prevalent DOAC users, and accounting for cancer duration and the competing risk of death (Table 3). No association was identified between the use of tamoxifen (9 of 1179 [0.8%]) or an AI (32 of 3574 [0.9%]) and the negative outcomes of cholecystitis, diverticulitis, and appendicitis (weighted HR, 0.89 [95% CI, 0.42-1.90]).

**Table 3.** Summary of Additional Analyses

Additional analyses	Outcome, weighted HR (95% CI)	
	Major hemorrhage	Any hemorrhage
Restricted to those with eGFR measures and added as a covariate	0.67 (0.39-1.16)	1.09 (0.74-1.61)
Limit follow-up to 90 d	0.83 (0.41-1.68)	1.07 (0.62-1.86)
New DOAC users	0.73 (0.42-1.29)	1.09 (0.71-1.66)
Prevalent DOAC users	0.63 (0.31-1.29)	1.00 (0.61-1.66)
Duration from cancer diagnosis added as a covariate	0.68 (0.44-1.05)	1.03 (0.75-1.43)
Death as competing risk	0.68 (0.37-1.25)	1.04 (0.66-1.65)

Abbreviations: DOAC, direct oral anticoagulant; eGFR, estimated glomerular filtration rate; HR, hazard ratio.

## Discussion

For individuals with breast cancer, concurrent use of tamoxifen with DOACs was postulated to increase DOAC levels and lead to an elevated risk of hemorrhage based on the report of tamoxifen’s pharmacokinetic data.^17,18,19^ In this population-level study of older adults (≥66 years) with breast cancer, we detected no evidence of a higher hemorrhage risk with concurrent use of a DOAC and tamoxifen compared with an AI. The results were consistent when we used a more liberal definition of hemorrhage, restricting to those with eGFR measures, limiting follow-up duration to the first 90 days, stratifying by start of tamoxifen or AIs first or a DOAC first, considering duration from cancer diagnosis, and using death as a competing risk. These findings suggest that concerns of a higher risk of clinically relevant hemorrhage with coadministration of tamoxifen and a DOAC for patients with breast cancer are unwarranted and should not influence DOAC use when indicated.

Randomized clinical trials to date^31,32^ were performed in a limited number of participants and excluded those patients receiving potentially interfering medications, thereby limiting the ability to detect important clinical effects of DDI. In this regard, large administrative database studies are ideally suited to investigate adverse drug reactions as part of postmarketing surveillance and often inform safe prescribing practices. Tamoxifen is reported as an inhibitor of CYP3A4 and P-gp pathways,^17,18,19,33^ leading to a potentially higher risk of hemorrhage when combined with DOACs. However, limited clinical data exist regarding outcomes in patients with breast cancer taking concurrent DOACs and hormonal agents. A small retrospective study of 48 patients with breast cancer receiving adjuvant endocrine therapy (50% tamoxifen and 50% AI) and DOACs (rivaroxaban, apixaban, or dabigatran) for AF reported 3 gastrointestinal tract hemorrhages, all in individuals receiving tamoxifen plus rivaroxaban.^20^ This study raised possible concerns of a clinically relevant DDI, which could alter prescription practices such as empirical change or avoidance of certain anticoagulants or anticancer therapies, as recommended by major guidelines or guidance statements.^7,8,33^ Unnecessary switching to alternate anticoagulants could be detrimental to patients owing to the increased burden with low-molecular-weight heparin injections or frequent laboratory monitoring with vitamin K antagonists and decreased persistence.^34^ In addition, hemorrhage risk concerns may lead to empirical underdosing of DOACs and an increased risk of thromboembolic complications.^35^ Our study showed no clinical evidence of a higher risk of bleeding with concurrent use of a DOAC and tamoxifen relative to an AI, reassuring prescribers of the safety of their use and dismissing the need to switch either DOAC or hormonal agents because of concerns related to hemorrhage. Our findings are consistent with another study in patients with cancer and AF,^36^ which reported no increased risk of major bleeding when comparing 147 users of tamoxifen plus a DOAC with users of a DOAC only (adjusted rate ratio, 0.94 [95% CI, 0.38-2.33]). Compared with that study,^36^ our present study includes a much larger number of concurrent tamoxifen and DOAC users (n = 1179), used AIs as active comparators, and included patients with breast cancer only to allow comparison within a more homogeneous population.

### Strengths and Limitations

To our knowledge, this is the largest study of its kind to investigate the potential DDIs in patients with breast cancer taking a concurrent DOAC and hormonal agents. Our study can provide reassurance to clinicians and inform safe prescription decisions about concurrent use of a DOAC and tamoxifen. We included all patients in Ontario who met eligibility criteria, which provides a high degree of external validity. In addition, Ontario’s health care databases have several strengths, including extremely well-recorded prescription drug use^21^ and little emigration from the province (<1% per year).^37^ Our findings remain consistent across multiple sensitivity analyses. Our 3.1% rate of major hemorrhage events during a median follow-up of 166 days is comparable to reported rates from meta-analyses of randomized clinical trials of cancer-associated thrombosis and from the international registry of patients with venous thromboembolism.^38,39^ In addition, our study revealed that hemorrhage should not be underappreciated in this population because the major hemorrhage rate of 29.2 per 1000 person-years was not low but was similar to rates for patients with severe kidney dysfunction (eGFR <15 mL/min/1.73 m^2^), a known population with a high risk of hemorrhage.^40^

Our study has limitations. Retrospective observational cohort studies are subject to residual confounding. We attempted to overcome this by demonstrating no difference in the risk for a dummy outcome that serves as a negative control. A further limitation is confounding by treatment indication that we attempted to minimize by comparing tamoxifen and AI users, both common therapies in breast cancer given to similar patient populations.^41,42,43^ Nevertheless, differences in baseline characteristics were noted between tamoxifen and AI users that were well balanced after weighting (Table 1 and eFigure 2 in the Supplement). Our study was limited to patients 66 years or older, and as such our findings may not represent a younger population with breast cancer, because older patients are at high hemorrhage risk due to comorbidities, polypharmacy, and falls. Conversely, all patients were postmenopausal, making the population more homogeneous. Measures of frailty and other potential confounders were not available in our data sets. Certain hemorrhage-associated medications are available without a prescription in Ontario (eg, aspirin, nonsteroidal anti-inflammatory drugs) and may not be completely captured in the database. Nonetheless, there is no reason to suspect differential use between the exposure and comparator groups. An issue specific to database studies is misclassification of outcomes.^44^ Based on a reabstraction method examining major hemorrhage diagnostic code validity in medical records, the sensitivity of our *ICD-10* coding algorithm was 93%, whereas the positive predictive value was 87%.^27^ There were few edoxaban users (n = 56), given the later approval and less frequent use in Ontario. The causes of death are not available in the database. Last, because this is administrative database research and only prescription information is available, the actual adherence is unknown and assumed.

## Conclusions

In this population-based cohort study of individuals 66 years or older with breast cancer, the hemorrhage risk was not higher with tamoxifen-DOAC combination compared with AI-DOAC. This is consistent in multiple additional analyses and suggests that concurrent use of tamoxifen and DOACs is safe when clinically indicated.

## Notes

Supplement. **eTable 1.** Description of Individual Databases Held at ICES **eTable 2.** Drug Identification Numbers Used to Define Exposure Groups **eTable 3.** Drug Identification Numbers of Strong CYP3A4 or P-gp Inhibitors as Exclusion **eTable 4.** Outcome Definition Codes **eFigure 1.** Cohort Flow Diagram **eFigure 2.** Density Plot Results of Comparison of Baseline Characteristics Between Tamoxifen and Aromatase Inhibitor Groups Before and After Propensity Score Weighting **eFigure 3.** Kaplan-Meier Curve of Major Hemorrhage Events Comparing Tamoxifen to Aromatase Inhibitor **eFigure 4.** Kaplan-Meier Curve of Any Hemorrhage Events Comparing Tamoxifen to Aromatase Inhibitor
