# Supplementary material for: Hemorrhage Risk Among Patients With Breast Cancer Receiving Concurrent Direct Oral Anticoagulants With Tamoxifen vs Aromatase Inhibitors
Source: JAMA Netw Open. 2022 Jun 28;5(6):e2219128. doi: 10.1001/jamanetworkopen.2022.19128 (PMC9240900; doi:10.1001/jamanetworkopen.2022.19128)
Supplement: Supplement. — eTable 1. Description of Individual Databases Held at ICES eTable 2. Drug Identification Numbers Used to Define Exposure Groups eTable 3. Drug Identification Numbers of Strong CYP3A4 or P-gp Inhibitors as Exclusion eTable 4. Outcome Definition Codes eFigure 1. Cohort Flow Diagram eFigure 2. Density Plot Results of Comparison of Baseline Characteristics Between Tamoxifen and Aromatase Inhibitor Groups Before and After Propensity Score Weighting eFigure 3. Kaplan-Meier Curve of Major Hemorrhage Events Comparing Tamoxifen to Aromatase Inhibitor eFigure 4. Kaplan-Meier Curve of Any Hemorrhage Events Comparing Tamoxifen to Aromatase Inhibitor [file jamanetwopen-e2219128-s001.pdf]

Supplementary Online Content

Wang TF, Clarke AE, Awan AA, Tanuseputro P, Carrier M, Sood MM. Hemorrhage risk among patients with breast cancer receiving concurrent direct oral anticoagulants with tamoxifen vs aromatase inhibitors. *JAMA Netw Open*. 2022;5(6):e2219128. doi:10.1001/jamanetworkopen.2022.19128

eTable 1. Description of Individual Databases Held at ICES

eTable 2. Drug Identification Numbers Used to Define Exposure Groups

eTable 3. Drug Identification Numbers of Strong CYP3A4 or P-gp Inhibitors as Exclusion

eTable 4. Outcome Definition Codes

eFigure 1. Cohort Flow Diagram

eFigure 2. Density Plot Results of Comparison of Baseline Characteristics Between Tamoxifen and Aromatase Inhibitor Groups Before and After Propensity Score Weighting

eFigure 3. Kaplan-Meier Curve of Major Hemorrhage Events Comparing Tamoxifen to Aromatase Inhibitor

eFigure 4. Kaplan-Meier Curve of Any Hemorrhage Events Comparing Tamoxifen to Aromatase Inhibitor

This supplementary material has been provided by the authors to give readers additional information about their work.

© 2022 Wang TF et al. *JAMA Network Open*.

eTable 1. Description of Individual Databases Held at ICES

**ICES Data Sources**
Ontario Drug Benefit (ODB) Database: The ODB formulary includes a wide range of routine outpatient medications, including oral preparations of the prescription drugs of interest to this study. The error rate in this database for drug and dose dispensed is minimal (~0.7%, 95% CI 0.5% to 0.9%). We used the ODB database to determine exposure to the cancer interfering drugs and to a direct oral anticoagulant (DOAC), including rivaroxaban, apixaban, edoxaban, and dabigatran. The date of first prescriptions of concurrent use of a DOAC and a cancer drug of interest was considered as start of the exposure period. We obtained information on each drug of interest including the drug identification number, trade name, therapeutic class, pill strength, quantity dispensed, days supplied, and formulations.
Canadian Institute of Health Information Discharge Abstract Database (CIHI-DAD): The CIHI-DAD collects diagnostic, and procedural variables for each admission to a hospital in Ontario. Coding of primary and secondary diagnoses and inpatient procedures uses the 9th version of the Canadian Modified International Classification of Disease system (ICD-9 CA) prior to 2002 and the 10th version (ICD-10 CA) for all diagnoses after 2002. We used the CIHI-DAD to assess hospital admissions with the primary outcome of hemorrhage. In addition, we used the CIHI-DAD to obtain demographics, assess hospitalizations prior to the index date and co-morbid conditions for each patient in the five years prior to the index date. These characteristics acted as study inclusion or exclusion criteria, or confounders in the multivariable models.
Ontario Health Insurance Plan (OHIP) Claims History Database: Most physicians in Ontario submit billing claims using fee and diagnosis codes outlined in the OHIP Schedule of Benefits. These codes capture information on inpatient, outpatient, and laboratory services rendered to a patient. In addition, OHIP includes information on the nature of the service and diagnostic information. Similar to the CIHI-DAD, these variables were used as covariates for overlap propensity score weighting for the exposure (interfering) drugs of interest. In chart re-abstraction studies, agreement between abstracted OHIP codes compared to the actual code recorded on the chart by the physician for the “most responsible” diagnosis was over 90% while percent agreement for procedural codes was over 88%.

© 2022 Wang TF et al. *JAMA Network Open*.

Registered Persons Database (RPDB): The RPDB captures information regarding Ontarians' gender, date of birth, postal code, and vital status.
**National Ambulatory Care Reporting System (NACRS):** The NACRS is compiled by the Canadian Institute for Health Information (CIHI) and contains administrative, clinical (diagnoses and procedures), demographic, and administrative information for all patient visits made to hospital- and community-based ambulatory care centres (emergency departments, day surgery units, hemodialysis units, and cancer care clinics) in Ontario. At ICES, NACRS records are linked with other data sources (DAD, Ontario Mental Health Reporting System [OMHRS]) to identify transitions to other care settings, such as inpatient acute care or psychiatric care. Prior to April 1, 2002, diagnoses (up to 6 on a given NACRS record) are captured using the ICD-9 coding system and procedures (up to 10 on a given NACRS record) are captured using the CCP coding system. Following April 1, 2002, diagnoses (up to 10 on a given NACRS record) are captured using the ICD-10-CA coding system and interventions (up to 10 on a given NACRS record) are captured using the CCI coding system. NACRS emergency department diagnosis codes have been extensively validated.
Ontario Laboratory Information System (OLIS): The Ontario Laboratory Information System (OLIS) is an electronic system that contains laboratory tests conducted for patients in Ontario. Data is available from 2007 with serum creatinine values cleaned and at ICES Central. In the database the number of individuals older than 66 years having at least one serum creatinine is greater than 3 million. This database allowed us to establish a subset of patients with chronic kidney disease defined by serum creatinine laboratory values and estimated glomerular filtration rates.
Ontario Cancer Registry (OCR): The OCR is a computerized database of information on all Ontario residents who have been newly diagnosed with cancer since 1964. All new cases of cancer, expect non-melanoma skin cancer, are registered in the information system which is managed and maintained by Cancer Care Ontario (CCO). Data from multiple sources, including DAD and SDS records from CIHI which include a diagnosis of cancer, paper reports from pathology departments with any mention of cancer, electronic reports from the eight Ontario Regional Cancer Centers and from the Princess Margaret Hospital (the specialized institutions treated cancer patients in Ontario), and electronic reports of all deaths of Ontario residents from the Office of the Registrar General of Ontario based on Ontario Provincial death certificates with cancer as the underlying cause of death are linked to compile incident cases of cancer in Ontario.

© 2022 Wang TF et al. *JAMA Network Open*.

Approximately 95% of all diagnosed cancer cases in Ontario are captured by the OCR. When using a clinical registry of head and neck tumours from a provincial regional cancer centre as the reference standard, there was excellent agreement with the OCR for tumor site (81%) and diagnosis date within 1 month (91.5%).

© 2022 Wang TF et al. *JAMA Network Open*.

eTable 2. Drug Identification Numbers Used to Define Exposure Groups

Drug	Drug Identification Number
Direct oral anticoagulant (DOAC)	02312441, 02358808, 02316986, 02378604, 02378612, 02377233, 02397714, 09857463, 02468905, 02468913, 02480808
Tamoxifen	00657379, 00657379, 00657379, 00657379, 00657360, 00657360, 00657360, 00657360, 01951947, 01951947, 01951947, 01951947, 01951939, 01951939, 01951939, 01951939, 01926632, 01926632, 01926632, 01926632, 01926624, 01926624, 01926624, 01926624, 02237460, 02237460, 02237460, 02237460, 02237459, 02237459, 02237459, 02237459, 99113709, 99113709, 99113709, 99113709, 02296748, 02296748, 02296748, 02296748, 02296748, 02296748, 02296748, 02296748, 02296748, 02296748, 02296748, 02296748, 02296748, 02296748, 02296748, 02296748, 02296721, 02296721, 02296721, 02296721, 02296721, 02296721, 02296721, 02296721, 02296721, 02296721, 02296721, 02296721, 02296721, 02296721, 02296721, 02296721, 02048485, 02048485, 02048485, 02048485, 02048485, 02048485, 02048485, 02048485, 02048485, 02048485, 02048485, 02048485, 02048485, 02048485, 02048485, 02048485, 02048477, 02048477, 02048477, 02048477, 02048477, 02048477, 02048477, 02048477, 02048477, 02048477, 02048477, 02048477, 02048477, 02048477, 02048477, 02048477, 02237599, 02237599, 02237599, 02237599, 02237599, 02237599, 02237599, 02237599, 02237599, 02237599, 02237599, 02237599, 02237599, 02237599, 02237599, 02237599, 02237598, 02237598, 02237598, 02237598, 02237598, 02237598, 02237598, 02237598, 02237598, 02237598, 02237598, 02237598, 02237598, 02237598, 02237598, 02237598, 02237597, 02237597, 02237597, 02237597, 02237597, 02237597, 02237597, 02237597, 02237597, 02237597, 02237597, 02237597, 02237597, 02237597, 02237597, 02237597, 02237596, 02237596, 02237596, 02237596, 02237596, 02237596, 02237596, 02237596, 02237596, 02237596, 02237596, 02237596, 02237596, 02237596, 02237596, 02237596, 02089858, 02089858, 02089858, 02089858, 02089858, 02089858, 02089858, 02089858, 02089858, 02089858, 02089858, 02089858, 02089858, 02089858, 02089858, 02089858, 02088428, 02088428, 02088428, 02088428, 02088428, 02088428, 02088428, 02088428, 02088428, 02088428, 02088428, 02088428, 02088428, 02088428, 02088428, 02088428, 02084430, 02084430, 02084430, 02084430, 02084430, 02084430, 02084430, 02084430, 02084430, 02084430, 02084430, 02084430, 02084430, 02084430, 02084430, 02084430, 02084422, 02084422, 02084422, 02084422, 02084422, 02084422, 02084422, 02084422,

© 2022 Wang TF et al. *JAMA Network Open*.

	02084422, 02084422, 02084422, 02084422, 02084422, 02084422, 02084422, 02084422, 02232552, 02232552, 02232552, 02232552, 02232552, 02232552, 02232552, 02232552, 02232552, 02232552, 02232552, 02232552, 02232552, 02232552, 02232552, 02232552, 02232551, 02232551, 02232551, 02232551, 02232551, 02232551, 02232551, 02232551, 02232551, 02232551, 02232551, 02232551, 02232551, 02232551, 02232551, 02232551, 02147467, 02147467, 02147467, 02147467, 02147467, 02147467, 02147467, 02147467, 02147467, 02147467, 02147467, 02147467, 02147467, 02147467, 02147467, 02147467, 02147459, 02147459, 02147459, 02147459, 02147459, 02147459, 02147459, 02147459, 02147459, 02147459, 02147459, 02147459, 02147459, 02147459, 02147459, 02147459, 02230043, 02230043, 02230043, 02230043, 02230043, 02230043, 02230043, 02230043, 02230043, 02230043, 02230043, 02230043, 02230043, 02230043, 02230043, 02230042, 02230042, 02230042, 02230042, 02230042, 02230042, 02230042, 02230042, 02230042, 02230042, 02230042, 02230042, 02230042, 02230042, 02230042, 02230042, 02063778, 02063778, 02063778, 02063778, 02063778, 02063778, 02063778, 02063778, 02063778, 02063778, 02063778, 02063778, 02063778, 02063778, 02063778, 02063778, 02063751, 02063751, 02063751, 02063751, 02063751, 02063751, 02063751, 02063751, 02063751, 02063751, 02063751, 02063751, 02063751, 02063751, 02063751, 02063751, 02220660, 02220660, 02220660, 02220660, 02220660, 02220660, 02220660, 02220660, 02220660, 02220660, 02220660, 02220660, 02220660, 02220660, 02220660, 02220660, 02220652, 02220652, 02220652, 02220652, 02220652, 02220652, 02220652, 02220652, 02220652, 02220652, 02220652, 02220652, 02220652, 02220652, 02220652, 02220652, 01982273, 01982273, 01982273, 01982273, 01982273, 01982273, 01982273, 01982273, 01982273, 01982273, 01982273, 01982273, 01982273, 01982273, 01982273, 01982273, 00911731, 00911731, 00911731, 00911731, 00911731, 00911731, 00911731, 00911731, 00911731, 00911731, 00911731, 00911731, 00911731, 00911731, 00911731, 00911731, 00911723, 00911723, 00911723, 00911723, 00911723, 00911723, 00911723, 00911723, 00911723, 00911723, 00911723, 00911723, 00911723, 00911723, 00911723, 00911723, 00911722, 00911722, 00911722, 00911722, 00911722, 00911722, 00911722, 00911722, 00911722, 00911722, 00911722, 00911722, 00911722, 00911722, 00911722, 00911722, 00911722, 00851973, 00851973, 00851973, 00851973, 00851973, 00851973, 00851973, 00851973, 00851973, 00851973, 00851973, 00851973, 00851973, 00851973, 00851973, 00851973, 00851965, 00851965,

© 2022 Wang TF et al. *JAMA Network Open*.

	00851965, 00851965, 00851965, 00851965, 00851965, 00851965, 00851965, 00851965, 00851965, 00851965, 00851965, 00851965, 00851965, 00851965, 00839362, 00839362, 00839362, 00839362, 00839362, 00839362, 00839362, 00839362, 00839362, 00839362, 00839362, 00839362, 00839362, 00839362, 00839362, 00839362, 00839361, 00839361, 00839361, 00839361, 00839361, 00839361, 00839361, 00839361, 00839361, 00839361, 00839361, 00839361, 00839361, 00839361, 00839361, 00839361, 00839353, 00839353, 00839353, 00839353, 00839353, 00839353, 00839353, 00839353, 00839353, 00839353, 00839353, 00839353, 00839353, 00839353, 00839353, 00839353, 00638706, 00638706, 00638706, 00638706, 00638706, 00638706, 00638706, 00638706, 00638706, 00638706, 00638706, 00638706, 00638706, 00638706, 00638706, 00638706, 00812404, 00812404, 00812404, 00812404, 00812404, 00812404, 00812404, 00812404, 00812404, 00812404, 00812404, 00812404, 00812404, 00812404, 00812404, 00812404, 00812390, 00812390, 00812390, 00812390, 00812390, 00812390, 00812390, 00812390, 00812390, 00812390, 00812390, 00812390, 00812390, 00812390, 00812390, 00812390, 00810452, 00810452, 00810452, 00810452, 00810452, 00810452, 00810452, 00810452, 00810452, 00810452, 00810452, 00810452, 00810452, 00810452, 00810452, 00810452, 00810444, 00810444, 00810444, 00810444, 00810444, 00810444, 00810444, 00810444, 00810444, 00810444, 00810444, 00810444, 00810444, 00810444, 00810444, 00810444, 00419052, 00419052, 00419052, 00419052, 00419052, 00419052, 00419052, 00419052, 00419052, 00419052, 00419052, 00419052, 00419052, 00419052, 00419052, 00419052
Anastrozole	02224135, 02313049, 02320738, 02328690, 02338467, 02339080, 02351218, 02361418, 02365650, 02374420, 02379104, 02379562, 02393573, 02394898, 02404990, 02417855, 02427818, 02458799
Letrozole	02231384, 02309114, 02322315, 02338459, 02343657, 02344815, 02347997, 02348969, 02358514, 02372169, 02372282, 02373009, 02373424, 02404400, 02421585, 02428156, 02459884
Exemestane	02242705, 02390183, 02407841, 02408473, 02419726

© 2022 Wang TF et al. *JAMA Network Open*.

eTable 3. Drug Identification Numbers of Strong CYP3A4 or P-gp Inhibitors as Exclusion

Drug	Drug Identification Number
Cyclosporin	9857771,9857774, 755591,755605, 1907182, 2150662, 2150670, 2150689, 2150697, 2237671, 2242821, 2244324, 2247073, 2247074, 9850473, 9852182, 9857097, 9857129, 9857176, 9857184, 9857192, 9857206, 9857214, 9857613, 9857614, 9857770
Tacrolimus	2175983, 2175991, 2176009, 2243144, 2244148, 2244149, 2296462, 2296470, 2296489, 2331667, 2416816, 2416824, 2416832, 9851984, 9852069, 9852662, 9854786
Itraconazole	2047454, 2231347, 2462559, 9857756
Ketoconazole	633836, 703974, 788813, 2231061, 2237235, 2245662
Voriconazole	2256460, 2256479, 2256487, 2279991, 2396866, 2396874, 2399245, 2399253, 2409674, 2409682, 9854663
Posaconazole	2293404, 2424622, 9900020
Quinine	311731, 704644, 26131, 4782, 21733, 23868, 26883, 94412, 249580, 346837, 441740, 1913883
Rifampin	210463, 210471, 343617, 393444, 580376, 580384, 2024861, 2091887, 2092808, 9900056

© 2022 Wang TF et al. *JAMA Network Open*.

eTable 4. Outcome Definition Codes

**Description**	**Code**	**Validation**
Major Hemorrhage	ICD10: I60 (excl I60.8), I61, I62, I85.0, I98.20, I98.3, K22.10, K22.12, K22.14, K22.16, K22.6, K22.8, K25.0, K25.2, K25.4, K25.6, K26.0, K26.2, K26.4, K26.6, K27.0, K27.2, K27.4, K27.6, K28.0, K28.2, K28.4, K28.6, K29.0, K31.80, K63.80, K92.0, K92.1, K55.20, K62.5, K92.2	Sensitivity: 94% (91-96), specificity: 83% (78-87), positive predictive value: 87% (83-90) and negative predictive value: 92% (88-95) (Arnason et al, 2006)
Any Hemorrhage	ICD10: I600, I601, I602, I603, I604, I605, I606, I607, I609, I61, I62, I850, I9820, I983, K2210, K2211, K2212, K2214, K2216, K226, K228, K250, K252, K254, K256, K260, K262, K264, K266, K270, K272, K274, K276, K280, K282, K284, K286, K290, K3180, K6380, K920, K921, K5520, K625, K922, N923, N938, N924, D699, R58, N020, N021, N022, N023, N024, N025, N026, N027, N028, N029, K661, N939, N950, R041, R042, R048, R049, R310, R311, R318, D683, H356, H431, H450, M250	
**Negative controls**		
Cholecystitis, diverticulitis, appendicitis	CIHI-DAD: K810, K811, K818, K819, K350, K351, K352, K353, K358, K359, K570, K571, K572, K57	

© 2022 Wang TF et al. *JAMA Network Open*.

eFigure 1. Cohort Flow Diagram

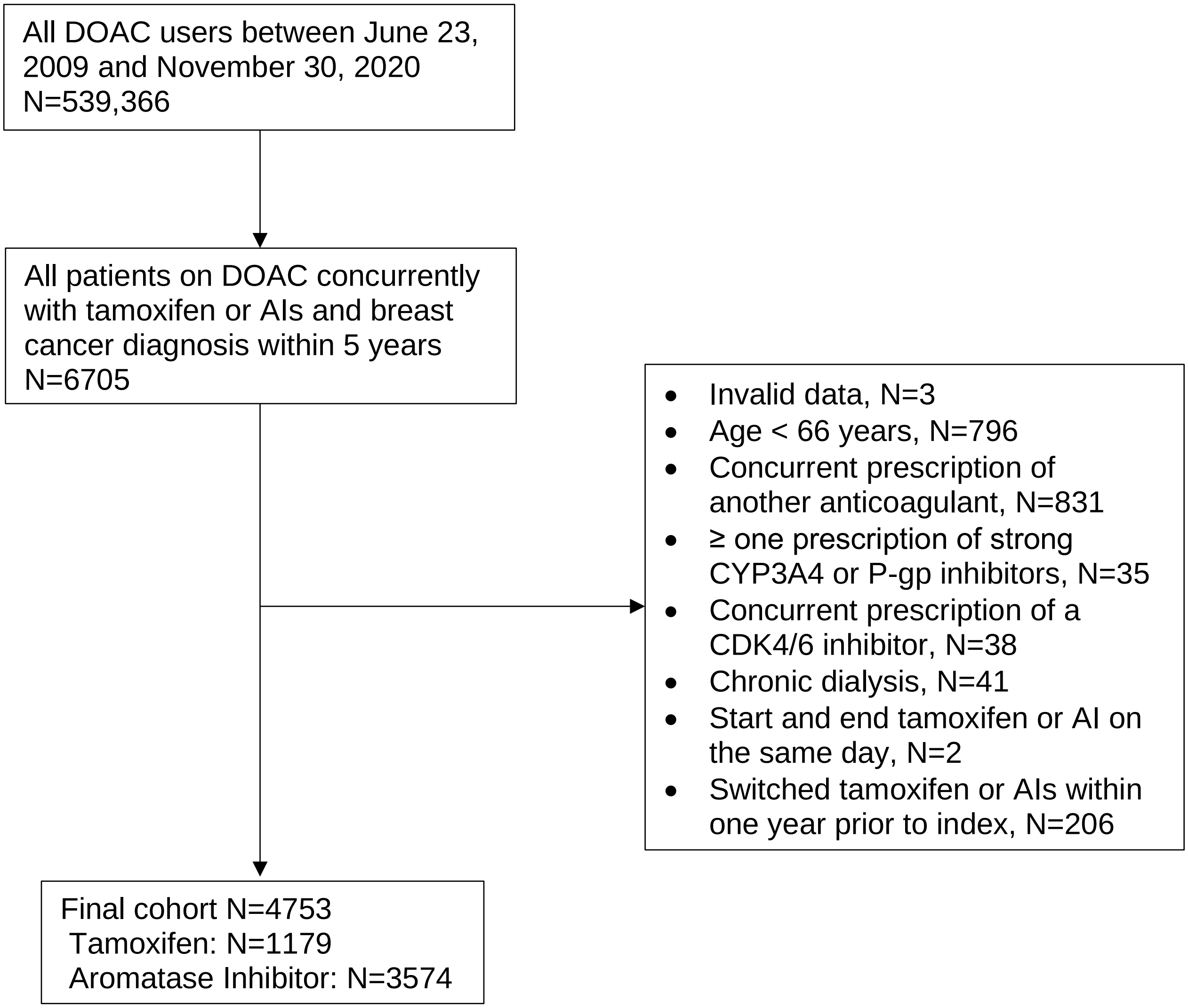

© 2022 Wang TF et al. *JAMA Network Open*.

eFigure 2. Density Plot Results of Comparison of Baseline Characteristics Between Tamoxifen and Aromatase Inhibitor Groups Before and After Propensity Score Weighting

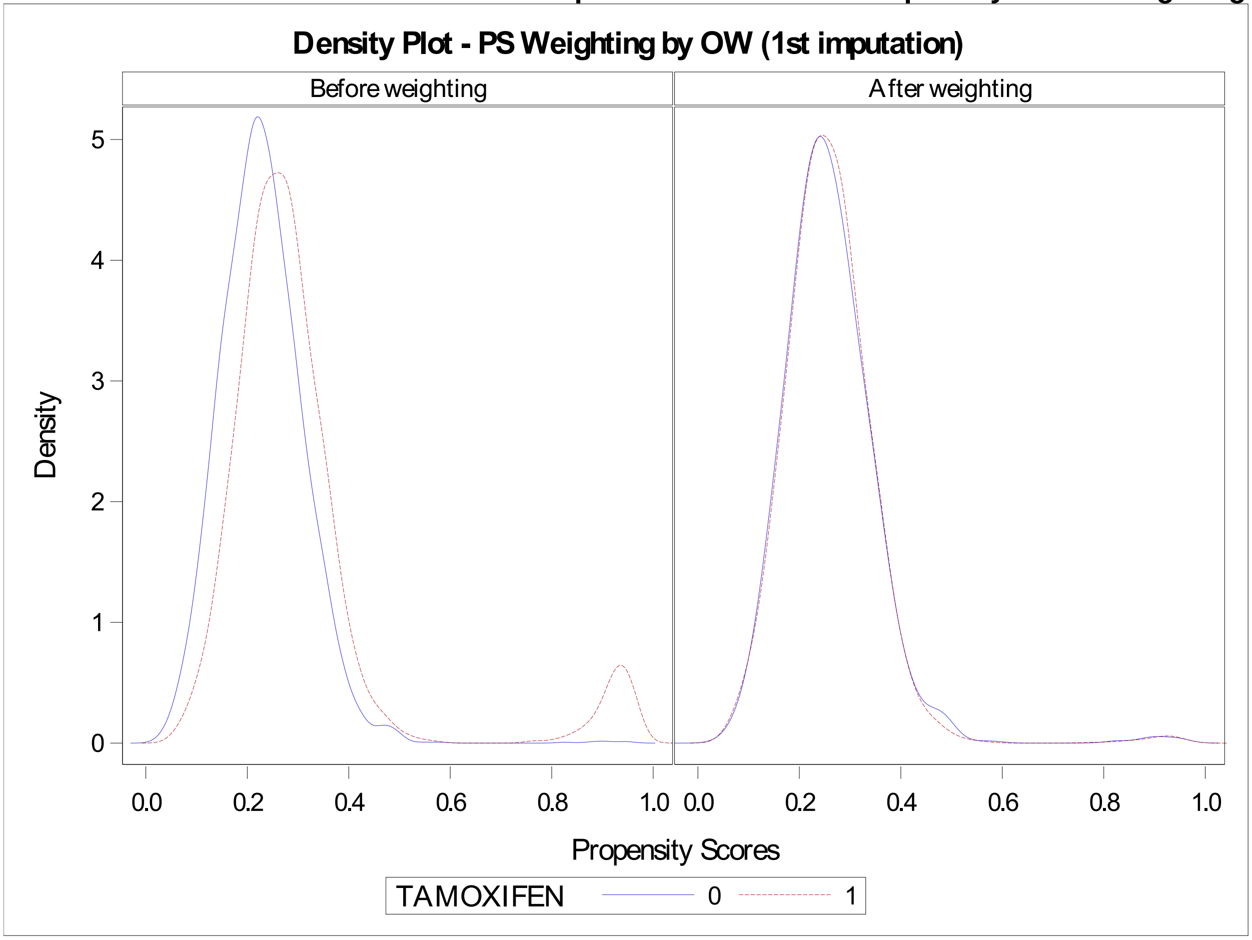

Analysis uses overlap weighting method (using first imputation as an example, data on other imputations are available upon request)

© 2022 Wang TF et al. *JAMA Network Open*.

eFigure 3. Kaplan-Meier Curve of Major Hemorrhage Events Comparing Tamoxifen to Aromatase Inhibitor

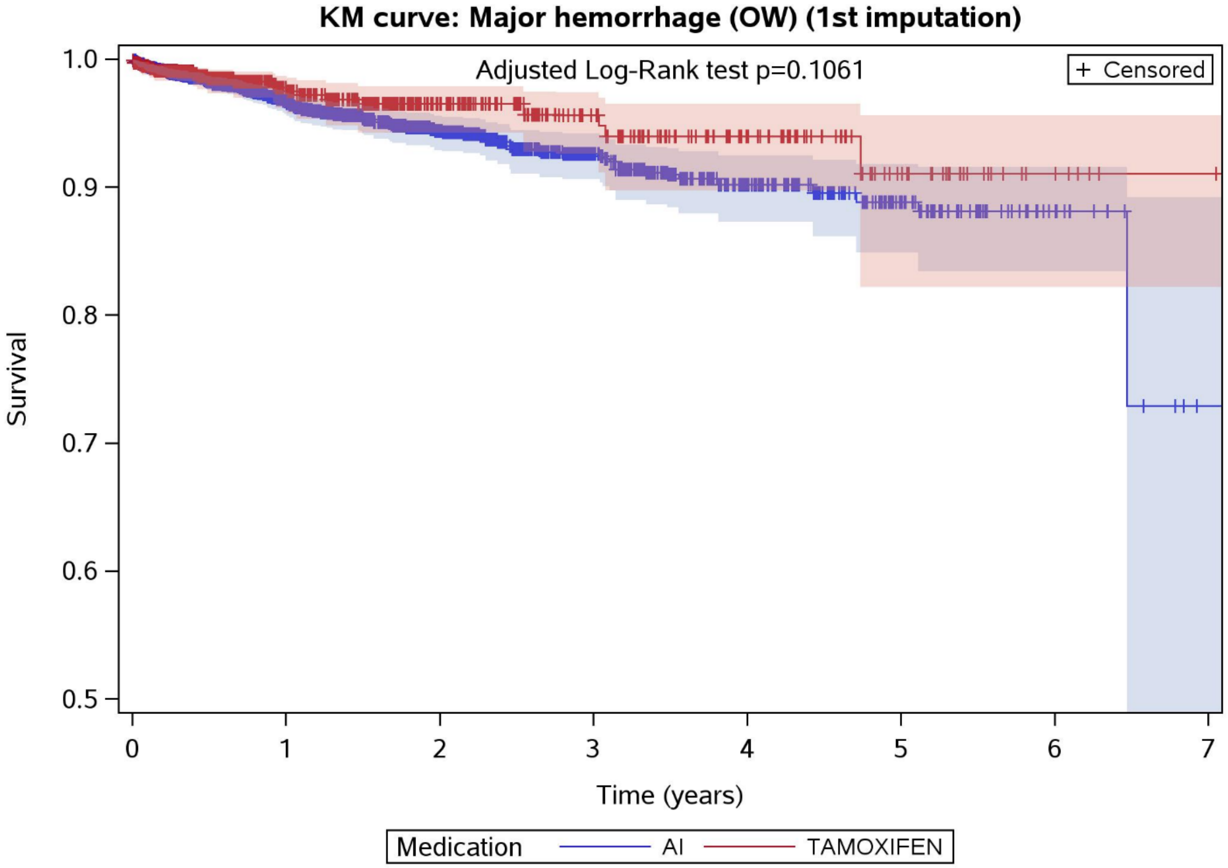

© 2022 Wang TF et al. *JAMA Network Open*.

eFigure 4. Kaplan-Meier Curve of Any Hemorrhage Events Comparing Tamoxifen to Aromatase Inhibitor

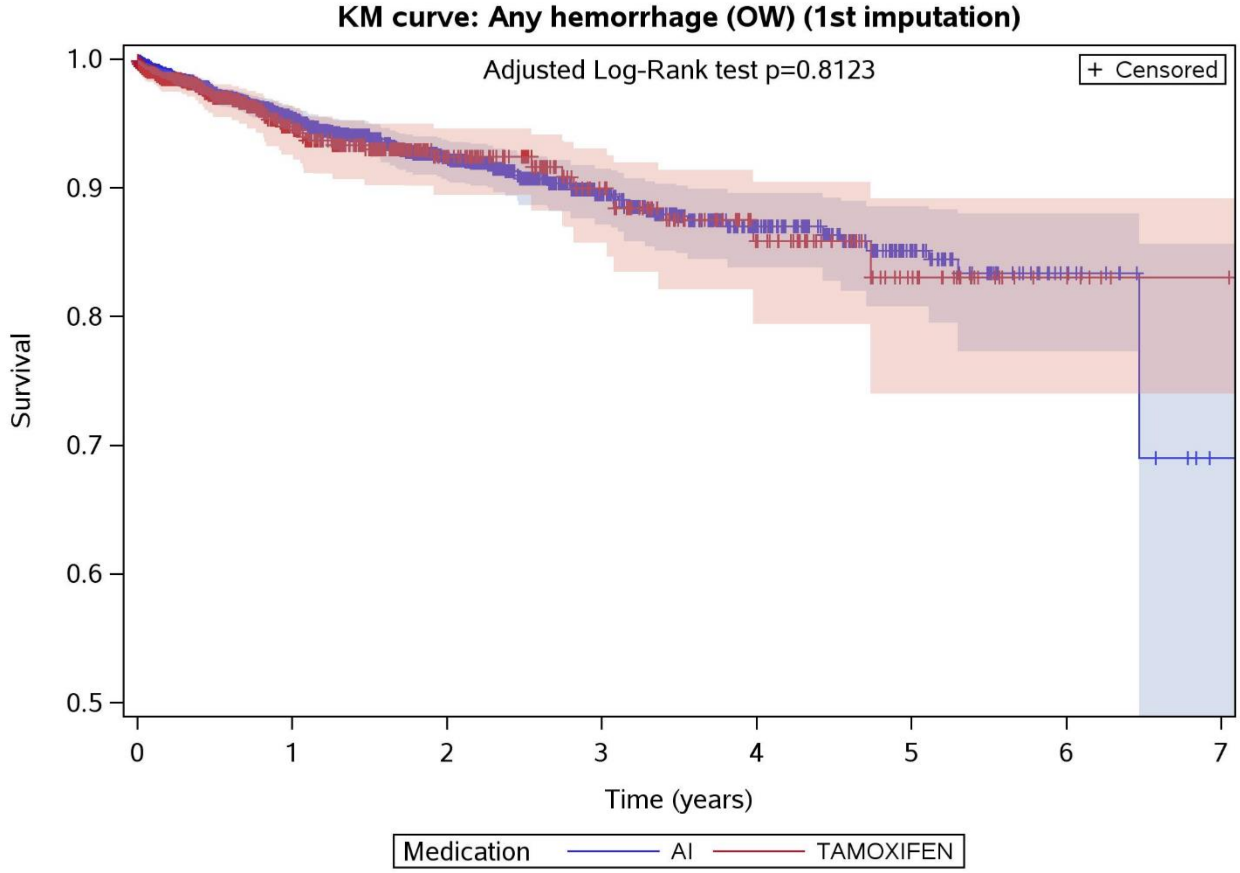

© 2022 Wang TF et al. *JAMA Network Open*.
